# Rethinking Omics Education in Brazil and South America: From Genomics to Multiomics and Critical Policy Studies

**DOI:** 10.1089/omi.2020.0064

**Published:** 2020-07-06

**Authors:** Alberto M.R. Dávila

**Affiliations:** Computational and Systems Biology Laboratory, Graduate Program in Biodiversity and Health, Oswaldo Cruz Institute, Fiocruz, Rio de Janeiro, Brazil.

## Perspective

Genomics has a storied past dating back to the 20th century, and impacted on discovery and translational science in medicine, ecology, and bioengineering, to mention but a few (Cuadrat et al., 2016). But the field of genomics has also been changing with the sands of time. Gone are the days when high-throughput technologies were limited to genomics. We now have multiomics discovery platforms including and beyond genomics such as metabolomics, proteomics, glycomics, among others (Gabius, 2018). Multiomics approach to integrative biology offers a fresh and exciting conceptual lens so as to triangulate data across the biological cascade from genes to proteins to metabolites and beyond. Such triangulation of data streams is at the core of contemporary multiomics systems science (Kunej, 2019).

Historically, applications of genomics have been varied ranging from personalized medicine to biotechnology products harnessed from the microbiome of extreme environments (Jorquera et al., 2019). These efforts are now being progressively expanded to the realm of multiomics inquiry. At the same time, such multipronged omics technology advances are highlighting the parallel need for omics education early in the course of the training of next-generation scholars, for example, with a focus on data science in undergraduate biology (Porter and Smith, 2019). Whitley et al. (2020) reviewed the importance of and needs for genomic education among academic, professional, and public platforms, thus underscoring the need to address the societal and educational dimensions of omics sciences that ought to extend beyond genomics.

In a context of genetics and genomics, the #GenomicDay initiative is spearheaded by the Computational and Systems Biology Laboratory of the Oswaldo Cruz Foundation (FIOCRUZ), a leading biomedical research institution in South America. This initiative was inspired by “Teaching the Genome Generation” developed by the Jackson Laboratory (LaRue et al., 2018; The Jackson Laboratory, 2020). In essence, #GenomicDay aims to encourage and engage broad public interest in genome sciences through critically informed science outreach and teaching the basic concepts of DNA, genes, and genomes and their various applications in health and society broadly.

Using accessible and relatable language, #GenomicDay seeks to reach out to high school students and teachers through a variety of communication strategies, such as lectures and creating hands-on formal and informal learning spaces. Because such initiatives engage science with scholars and high school students in an “upstream” formative early age and context, they conjure up new vistas and help imagine broadly framed multiple possible professional trajectories and creative careers in science and society in young minds.

The first edition of the #GenomicDay was successfully organized in October 2016 in Rio de Janeiro (FIOCRUZ, 2016). Owing to interest and relevance for high school students, additional #GenomicDay editions were annually organized in several regions of Brazil. The fifth edition initially planned for late 2020 will involve at least 20 high schools distributed nationwide representing the five regions of Brazil; for the first time, on-site sequencing will be carried in schools using sequencing technologies.

Because of the current Coronovirus Disease-2019 (COVID-19) pandemic, a need to properly inform society about omics and multiomics approaches in use for research on Severe acute respiratory syndrome coronovirus 2 (SARS-CoV-2), clinical studies, drugs, and vaccines was identified as a priority. Hence, a blog site akin to a science journalism and knowledge translation effort, containing short summaries using accessible and relatable language was created and named #GenomicNews (http://genomicnews.biowebdb.org).

After 4 years of organizing #GenomicDay, we are thus finally prepared to offer a complementary state-of-the-art initiative, one short-term hands-on course directed to high school teachers, communication and social media professionals, and physicians: #GenomicWeek. The goal of the latter initiative is to help knowledge mobilization for the concepts of DNA, genes, and genomes and their different application and impacts in society among professionals.

Looking further, it is conceivable that #GenomicDay, #GenomicNews, and #GenomicWeek can usefully serve as the home base for genomics and multiomics citizen science initiatives in Brazil, including and enhancing interactions with similar initiatives elsewhere in South America. For example, in 2018, the “1000 Genomas Chile” (http://www.1000genomas.cl/concurso2019) selected 10 high schools in Chile to sequence the genome of an Oniscidea species, to build a regional network for knowledge mobilization in genomics.

As the field has now evolved into multiomics science, an urgent need to scale up such knowledge mobilization efforts beyond genomics has become palpable. I propose that omics systems science stands to benefit, as with Brazil and South America, by extending the above vision on science education and outreach to a broader multitechnology context.

Still, science and technology outreach, alone, is not sufficient to cultivate critical thinking skills essential for any successful career in science and society (Fisher et al., 2010). One concept that holds vast potential but has not yet been enacted upon is the idea that science careers also include those in technology and innovation policy. Students in high school can benefit from learning the career options available to them in science beyond the laboratory space that impacts upon the broader society such as technology and innovation policy, but in ways that are critically informed (Sarewitz, 2016; Sclove, 2020).

That is, innovation policy requires thinking not only of scientific knowledge but also of epistemology of knowledge, that is, *how do we know what we know*? As students learn introductory concepts in science and technology policy, they can then begin to appreciate that how we frame a given field of research (i.e., epistemology) matters greatly for both laboratory work and science policy (Frodeman, 2020). For example, and as noted earlier in this perspective, framing systems science through genomics or multiomics lens determines whether and to what extent we incorporate complementary or alternative technologies relevant to answer the questions we pose in science.

The field of critical policy studies aims to address precisely these sorts of questions and helps cultivate a culture of curiosity and scientific inquiry that seeks out not only new scientific knowledge but also questions how that knowledge is framed in the first place (Von Schomberg, 2019). By shifting our attention in science education and outreach to a broader and more critical realm as already noted, technology and innovation policy in medicine, biology, and ecology in Brazil and South America can be harnessed more efficiently, and in ways attuned to broader societal values, by engaging with multiomics science education and the field of critical policy studies (Frodeman, 2020; Sclove, 2020; Von Schomberg, 2011; Von Schomberg and Hankins, 2019).
